# Effect of RIP Overexpression on Abiotic Stress Tolerance and Development of Rice

**DOI:** 10.3390/ijms22031434

**Published:** 2021-02-01

**Authors:** Pieter Wytynck, Jeroen Lambin, Simin Chen, Sinem Demirel Asci, Isabel Verbeke, Jeroen De Zaeytijd, Kondeti Subramanyam, Els J.M. Van Damme

**Affiliations:** 1 Laboratory of Biochemistry and Glycobiology, Department of Biotechnology, Ghent University, Coupure Links 653, 9000 Ghent, Belgium; Pieterwytynck@hotmail.com (P.W.); jeroenlambin@gmail.com (J.L.); simin.chen@ugent.be (S.C.); sinem.demirelasci@ugent.be (S.D.A.); Isabel.Verbeke@UGent.be (I.V.); jeroendezaeytijd@hotmail.com (J.D.Z.); prasamshika2@gmail.com (K.S.); 2 Center for Advanced Light Microscopy, Ghent University, 9000 Ghent, Belgium

**Keywords:** abiotic stress, localization, *Oryza sativa*, ribosome-inactivating protein, rice

## Abstract

Ribosome-inactivating proteins (RIPs) are a class of cytotoxic enzymes that can inhibit protein translation by depurinating rRNA. Most plant RIPs are synthesized with a leader sequence that sequesters the proteins to a cell compartment away from the host ribosomes. However, several rice RIPs lack these signal peptides suggesting they reside in the cytosol in close proximity to the plant ribosomes. This paper aims to elucidate the physiological function of two nucleocytoplasmic RIPs from rice, in particular, the type 1 RIP referred to as OsRIP1 and a presumed type 3 RIP called nuRIP. Transgenic rice lines overexpressing these RIPs were constructed and studied for developmental effects resulting from this overexpression under greenhouse conditions. In addition, the performance of transgenic seedlings in response to drought, salt, abscisic acid and methyl jasmonate treatment was investigated. Results suggest that both RIPs can affect methyl jasmonate mediated stress responses.

## 1. Introduction

Ribosome-inactivating proteins (RIPs) are enzymes capable of irreversible modification of ribosomal RNAs. RIPs are classified as RNA N-glycosidases (EC 3.2.2.22), which remove an adenine from a conserved loop, called the sarcin/ricin loop of rRNA [1,2,3]. This enzymatic activity renders the ribosome non-functional through the impaired interaction with elongation factor 2 [4]. Most RIPs studied so far are synthesized following the secretory route. However, cereals contain a large number of RIPs that do not contain a signal peptide [5]. The presence of RIPs in the cytoplasm (the same compartment that harbors the conspecific ribosomes) indicates that these RIPs might influence the plant itself. Some experiments have suggested that cereal RIPs may play a role in abiotic stress tolerance, next to a presumed role in plant defense [5]. Based on genome-wide searches, the RIP family in rice consists of type 1 RIPs (consisting of a single RIP domain) and potential type 3 RIPs (containing a RIP domain linked to a non-lectin domain) [6]. Of all RIP sequences identified in the rice genome, 69% do not contain a signal peptide sequence. Therefore, the corresponding proteins will be synthesized on the free ribosomes. At present, the function of rice RIPs is still enigmatic. Quantitative real-time polymerase chain reaction (qRT–PCR) and RNA sequencing data have shown that rice RIPs are synthesized in response to both abiotic and biotic stresses [6,7]. Overexpression of OSjRIP7.2 (LOC_Os07g37090) in rice conferred resistance to drought and salt stress [8]. Ding et al. [9] reported that a secreted RIP, OsjRIP7.2, is specifically expressed in developing anthers. The authors hypothesize that this RIP kills tapetal cells during the development of pollen [9].

In this study, we focus on two RIPs from rice that are synthesized without a signal peptide. OsRIP1 (OsjRIP1.1, LOC_Os01g06740) was identified recently and was shown to represent a nucleocytoplasmic type 1 RIP with proven enzymatic activity [10]. Based on RNA sequencing data, OsRIP1 expression was upregulated when rice seedlings were exposed to abscisic acid (ABA), jasmonate, cold, cadmium, drought and osmotic stress, while downregulation of OsRIP1 expression was observed when seedlings were exposed to flooding. The sequence of nuRIP (OsjRIP11.2, LOC_Os11g06460) has a high degree of similarity to the type 3 RIP from barley, called JIP60. NuRIP expression is upregulated after drought, flooding and osmotic stress but is downregulated after cadmium treatment [6]. Similar to OsRIP1, nuRIP also locates in the cytosol and the nucleus. This article aims to unravel the importance of two RIPs, in particular OsRIP1 and nuRIP, for the growth of rice when subjected to abiotic stresses and plant hormones. Transgenic lines overexpressing the RIPs were constructed, and their performance was evaluated under greenhouse conditions. In addition, the growth of transgenic seedlings was compared to the growth of wild-type plants when exposed to drought, salt, abscisic acid (ABA) and methyl jasmonate (MeJA).

## 2. Results

### 2.1. OsRIP1 and nuRIP Sequences, and Subcellular Localization

**Figure 1:**
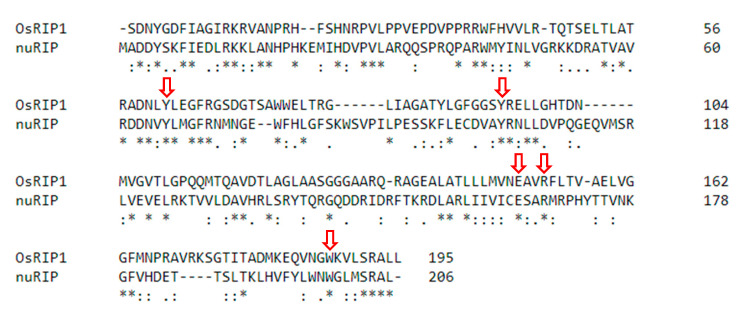
Alignment between the amino acid residues from ribosome-inactivating protein (RIP) domains of OsRIP1 and nuRIP. Red arrows point towards the active site residues for the RIP domain. Asterisks refer to residues that are fully conserved, colons indicate residues with highly similar properties, dots indicate residues with weakly similar properties.

**Figure 2:**
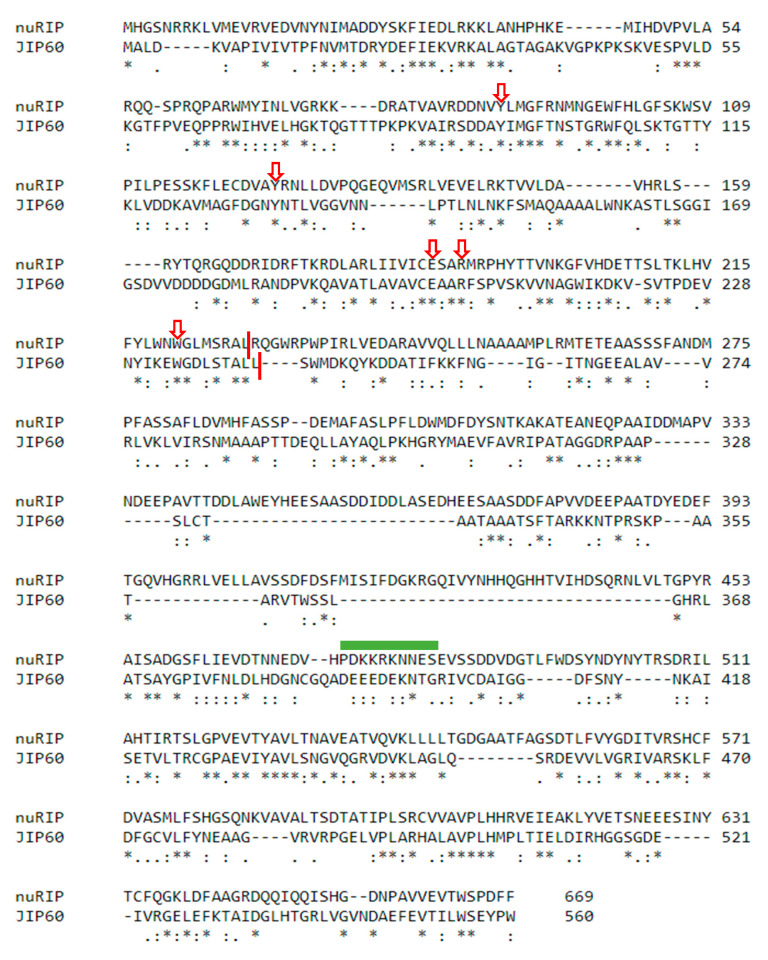
Alignment between the amino acid sequences for JIP60 and nuRIP. Red arrows point towards the active site residues for the RIP domain. The green bar shows the location of the nuclear localization signal in nuRIP. The vertical red lines indicate the end of the RIP domain for the two RIPs. Asterisks refer to residues that are fully conserved, colons indicate residues with highly similar properties, dots indicate residues with weakly similar properties.

Two RIP sequences from rice were selected for detailed analyses (Figure 1 and Figure 2). The OsRIP1 sequence encodes a 282 amino acid polypeptide lacking a signal peptide. Residues 18–210 are recognized as a RIP domain, encoding a type 1 RIP. In contrast, the nuRIP sequence contains an open reading frame encoding a polypeptide of 669 amino acids. Using NCBI’s conserved domain database, a RIP domain (amino acids 24–227) is recognized at the N-terminus of the nuRIP sequence. The C-terminal domain of the nuRIP sequence does not show homology to any known protein domain. Based on the presence of a RIP domain and the length of the polypeptide, nuRIP probably encodes a type 3 RIP. Sequence analyses revealed 27% sequence identity and 43% sequence similarity between the RIP domains from OsRIP1 and nuRIP. The active site residues (Y, Y, E, R, W) known to be important for RIP activity are identical in both sequences (Figure 1).

NuRIP shows an important degree of sequence similarity with the type 3 RIP JIP 60. The RIP domains from nuRIP and JIP60 show 31% sequence identity (45% sequence similarity), whereas the C-terminal sequences from nuRIP and JIP60 show 29% sequence identity (43% sequence similarity) (Figure 2).

**Figure 3:**
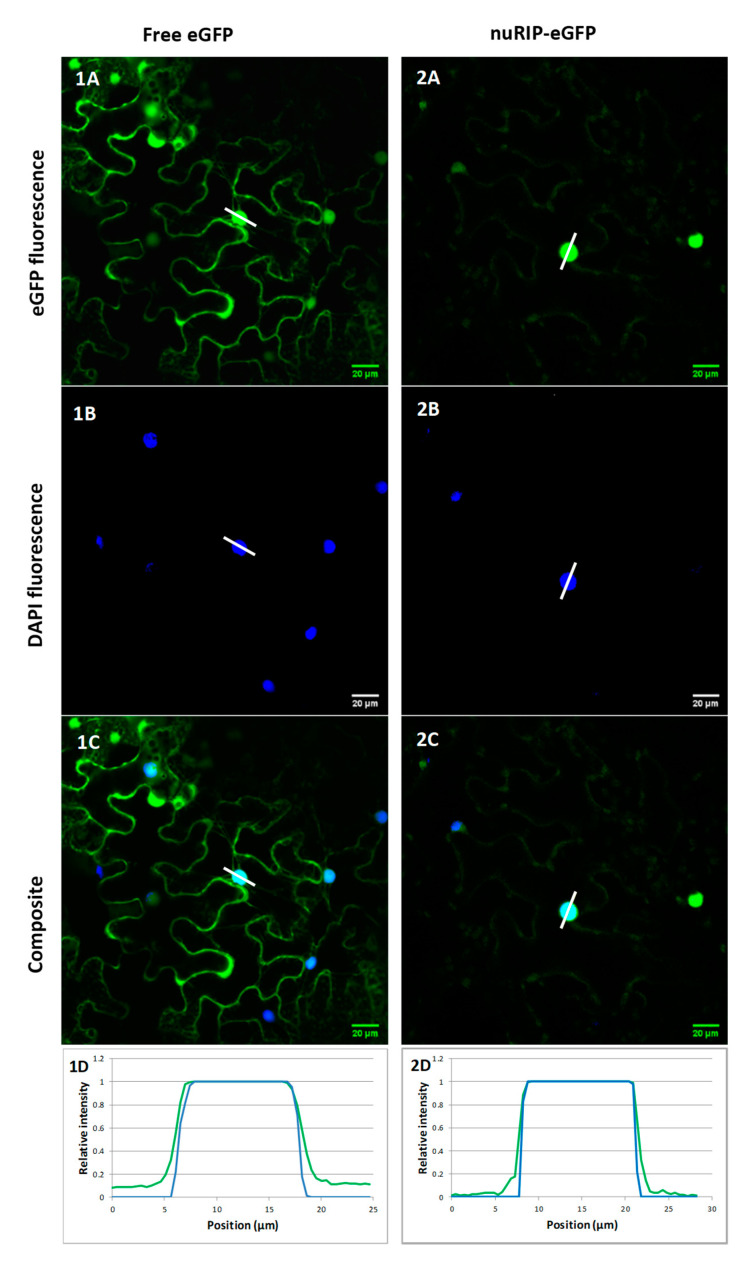
Subcellular localization of free eGFP construct (**left**) and nuRIP-eGFP fusion construct (**right**); The eGFP fluorescence is shown in panels 1–2A. The nuclei were stained with 4′, 6-diamidino-2-phenylindole (DAPI) in panels 1–2B. Composite images of both channels are shown in panels 1–2C. Panels 1–2D show the relative intensities of DAPI (blue) and eGFP (green) along the white lines in 1–2A, 1–2B and 1–2C. The y-axis represents the normalized fluorescence, while the x-axis represents the distance along the white line.

**Figure 4:**
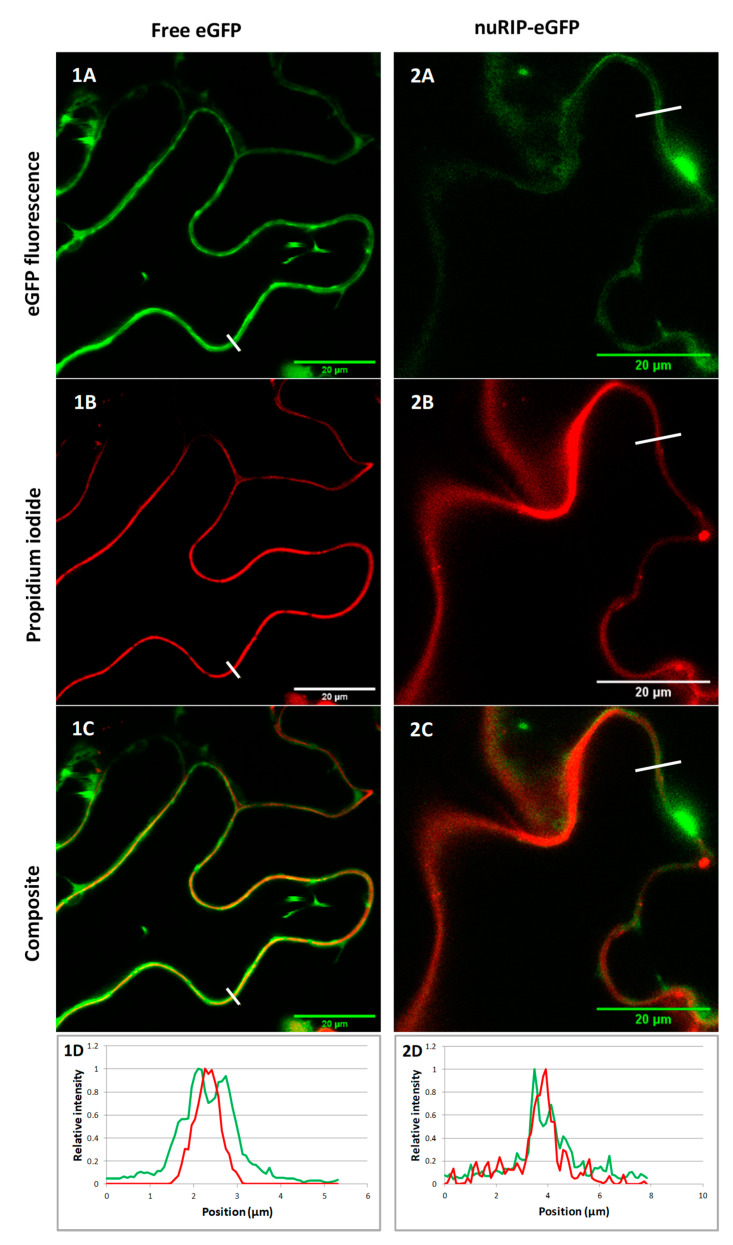
Zoom in of subcellular localization of free eGFP construct (**left**) and nuRIP-eGFP fusion construct (**right**). The eGFP fluorescence is shown in panels 1–2A. The cell wall was stained with propidium iodide in panels 1–2B. Composite images of both channels are shown in panels 1–2C. Panels 1–2D show the relative intensities of propidium iodide (red) and eGFP (green) along the white lines in 1–2A, 1–2B and 1–2C. The y-axis represents the normalized fluorescence, while the x-axis represents the distance along the white line.

Bioinformatics tools predicted that nuRIP contains no signal peptide but contains a nuclear localization signal at position 474 (PDKKRKNNES). The subcellular localization of nuRIP was studied by the transient transformation of tobacco leaf cells and compared to the localization of OsRIP1 that was recently shown to reside in the nucleus and cytosolic compartment of tobacco cells [10]. Colocalization studies with 4′, 6-diamidino-2-phenylindole (DAPI) and propidium iodide allowed to stain the nucleus and the cell wall, respectively. Fluorescence of fusion constructs for nuRIP coupled to enhanced green fluorescent protein (eGFP) was detected in the nucleus and the cytoplasm of tobacco cells (Figure 3). Identical results were obtained for N- and C-terminal fusion constructs. The eGFP fluorescence peak for the nuRIP fusion construct did not coincide with the fluorescence peak from propidium iodide, indicating that nuRIP does not localize to the cell wall (Figure 4).

### 2.2. Overexpression Lines for OsRIP1 and nuRIP

Transgenic lines overexpressing OsRIP1 and nuRIP using the constitutive ubiquitin promoter were created in *Oryza sativa* ssp. Japonica cv. Nipponbare. For each RIP construct, multiple independent transgenic lines were generated. Because of the relatively low amount of seeds obtained in the T1 generation, 2 or 3 lines were selected for seed propagation. All stress experiments were performed with T2 and T3 generation seeds.

Transcript levels for the RIPs were analyzed in shoots of 10-day old rice seedlings of the T2 and T3 generation. OsRIP1 expression levels in transgenic lines 1 and 2 were, respectively, 21-fold and 31-fold higher compared to wild-type plants. In plants of the T3-generation of line 1, a 14-fold higher expression of OsRIP1 was obtained compared to wild-type plants. Transgenic lines 1 and 2 (both T3-generation) overexpressing nuRIP reached overexpression levels of 14-fold and 3871-fold, respectively. The transcript levels for OsRIP1 and nuRIP in transgenic lines and wild-type plants are shown in Supplementary Figure S1.

### 2.3. Phenotypic Analysis of Transgenic Rice Lines

Phenotypic analysis was performed for the transgenic lines overexpressing OsRIP1 and nuRIP. The growth of two independent transgenic lines for each RIP was analyzed under greenhouse conditions and compared to the growth of wild-type plants. Plant length was measured at different growth stages, including the seedling stage (18 and 30 days after germination), the vegetative phase (50 days after germination), the reproductive phase (78 days after germination) and after ripening of the seeds (119 days after germination).

**Figure 5:**
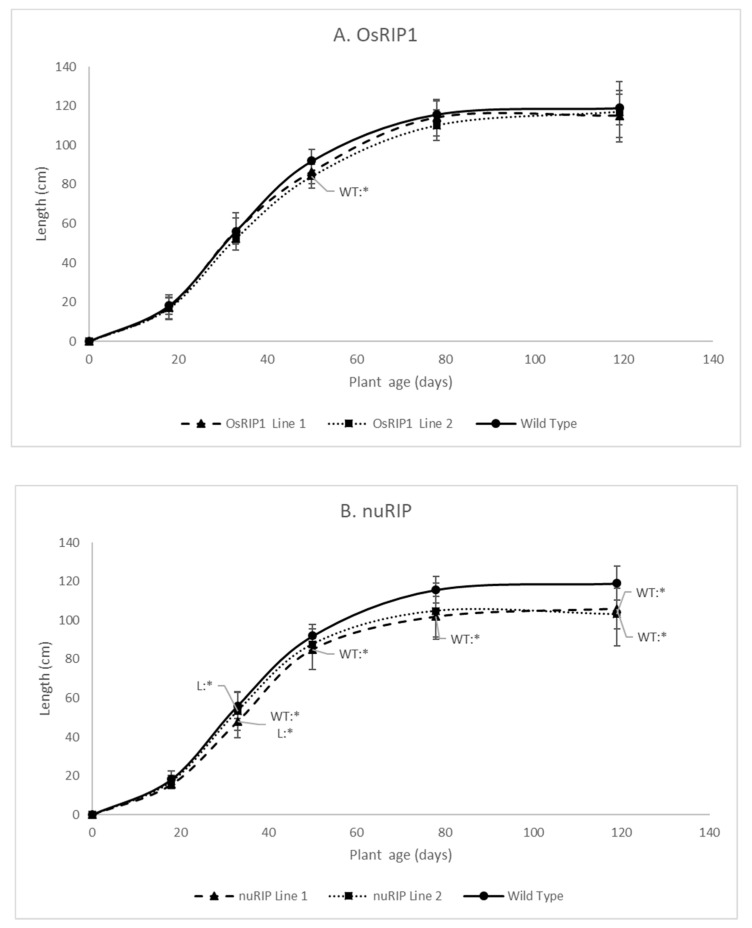
Growth curves from transgenic lines overexpressing OsRIP1 (panel **A**) or nuRIP (panel **B**) and wild-type plants at five different time points. The significant differences are labeled with stars at the different time points. WT:* refers to a significant difference between the transgenic line and the wild-type plants. L:* indicates a significant difference between the transgenic lines. Median values and interquartile range (error bars) are represented (data for each time point are based on 15 plants).

Transgenic lines overexpressing OsRIP1 showed a plant height that was similar to wild-type plants (Figure 5A). Plants from transgenic line 2 were significantly shorter at 50 days after germination, but these plants recovered towards the later time points. The plants overexpressing nuRIP were significantly shorter than wild-type plants, especially at the later time points during development (Figure 5B).

The slight growth retardation observed at the earlier time points for OsRIP1 overexpressing plants did not translate into a significant delay in flowering. In general, plants from nuRIP line 2 flowered earlier compared to other transgenic lines and wild-type plants. However, due to high variability between plants, no statistically significant differences were observed. Similarly, no significant differences were present in the 100-seed weight and the shoot mass at the end of the experiment.

### 2.4. Stress Tolerance of Transgenic Lines Overexpressing RIPs

Stress treatments were performed for 50 individual one-week-old seedlings of the transgenic lines overexpressing OsRIP1 or nuRIP. All the different treatments (ABA, MeJA, PEG (polyethylene glycol) and salt) used in this study inhibited the growth of the rice seedlings. After the stress treatment, the overall mass, the root and shoot length of wild-type plants were reduced (Supplementary Figure S2). The addition of MeJA showed the largest reduction in mass, root and shoot length in wild-type plants.

**Figure 6:**
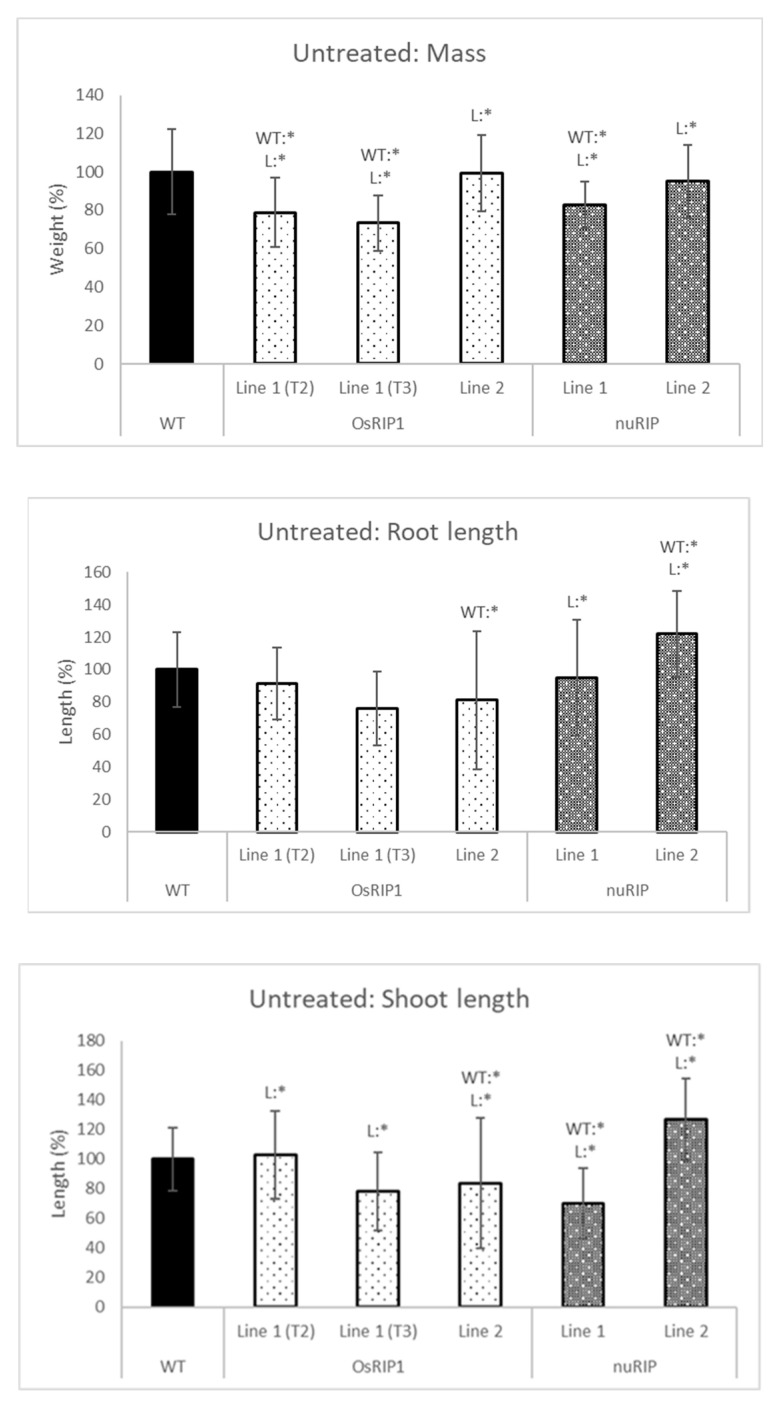
Total mass, shoot and root length of one-week-old plants from different transgenic lines compared to wild-type plants. WT:* refers to a significant difference to wild-type plants. L:* indicates a significant difference to the other transgenic line of the same construct. The histogram is based on averages; error bars represent the standard deviation. Each data point is gathered from 50 plants per line.

Seedlings from the different transgenic lines were grown side-by-side with wild-type plants in the greenhouse under controlled conditions for temperature and light (Figure 6). Total mass, root and shoot length were compared for plants grown in the absence of any stress factor. Seedlings belonging to OsRIP1 line 1 (both T2 and T3 generation seeds) yielded a significantly lower mass compared to wild-type plants. Seedlings from line 2 had a significantly shorter root and shoot. Although a similar trend was observed for line 1, the data were not significantly different from wild-type seedlings. Seedlings from nuRIP line 1 showed a lower mass than wild-type plants and had a lower shoot length. Although the overall mass of the plants from nuRIP line 2 was similar to that of wild-type plants, they had significantly longer root and shoot compared to wild-type plants.

**Figure 7:**
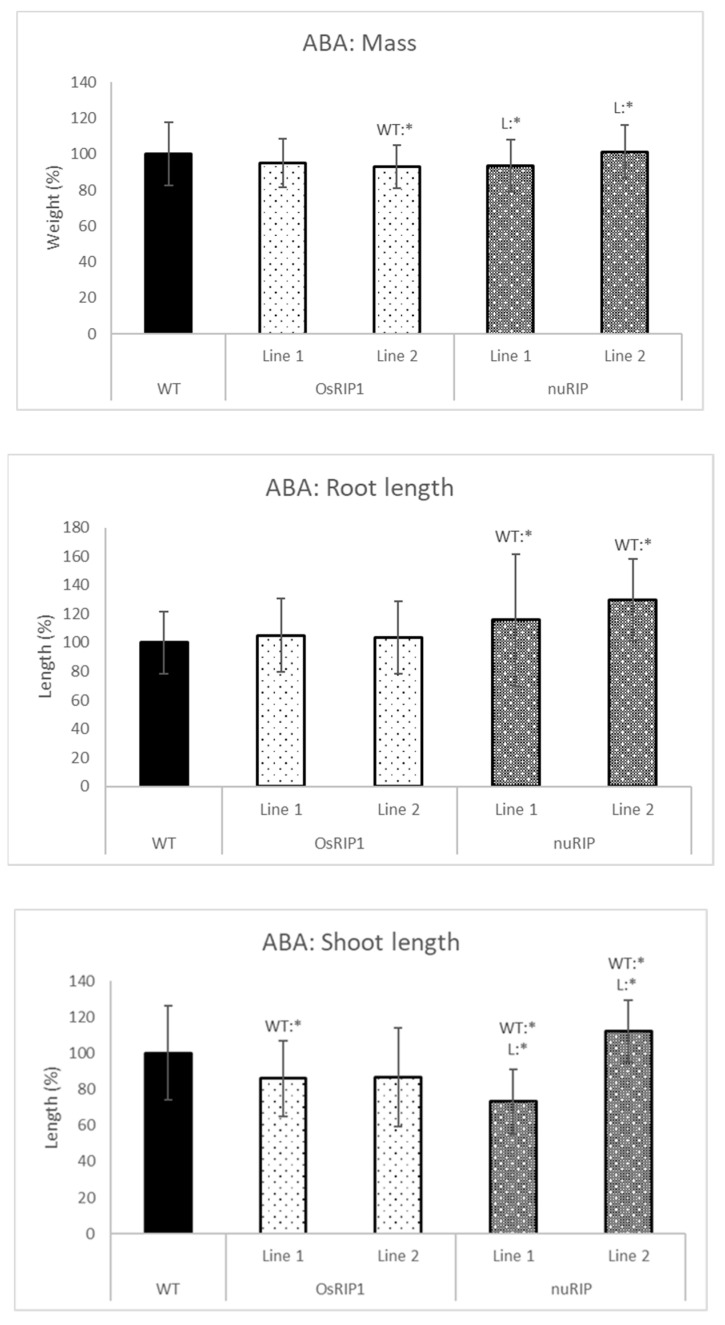
Total mass, shoot and root length of one-week-old plants from different transgenic lines subjected to 2 µM abscisic acid (ABA) compared to wild-type plants. WT:* refers to a significant difference to wild-type plants. L:* indicates a significant difference to the other transgenic line of the same construct. The histogram is based on averages; error bars represent the standard deviation. Each data point is gathered from 50 plants per line.

In addition, seedlings from transgenic lines and wild-type plants were subjected to hormone treatments with ABA and MeJA. Seedlings were treated with ABA by adding 2 µM ABA to the solution in which the seedlings were grown (Figure 7). No remarkable differences were observed in the growth of the transgenic lines overexpressing OsRIP1 compared to the wild-type plants after ABA treatment. Seedlings from line 2 revealed a lower mass, and plants from line 1 had a shorter shoot compared to wild-type seedlings. Contrasting results were obtained for the two lines overexpressing nuRIP. Seedlings from line 1 had a lower shoot length, while those from line 2 clearly had a longer shoot when compared to wild-type plants. Both nuRIP lines yielded longer roots than the wild-type plants.

**Figure 8:**
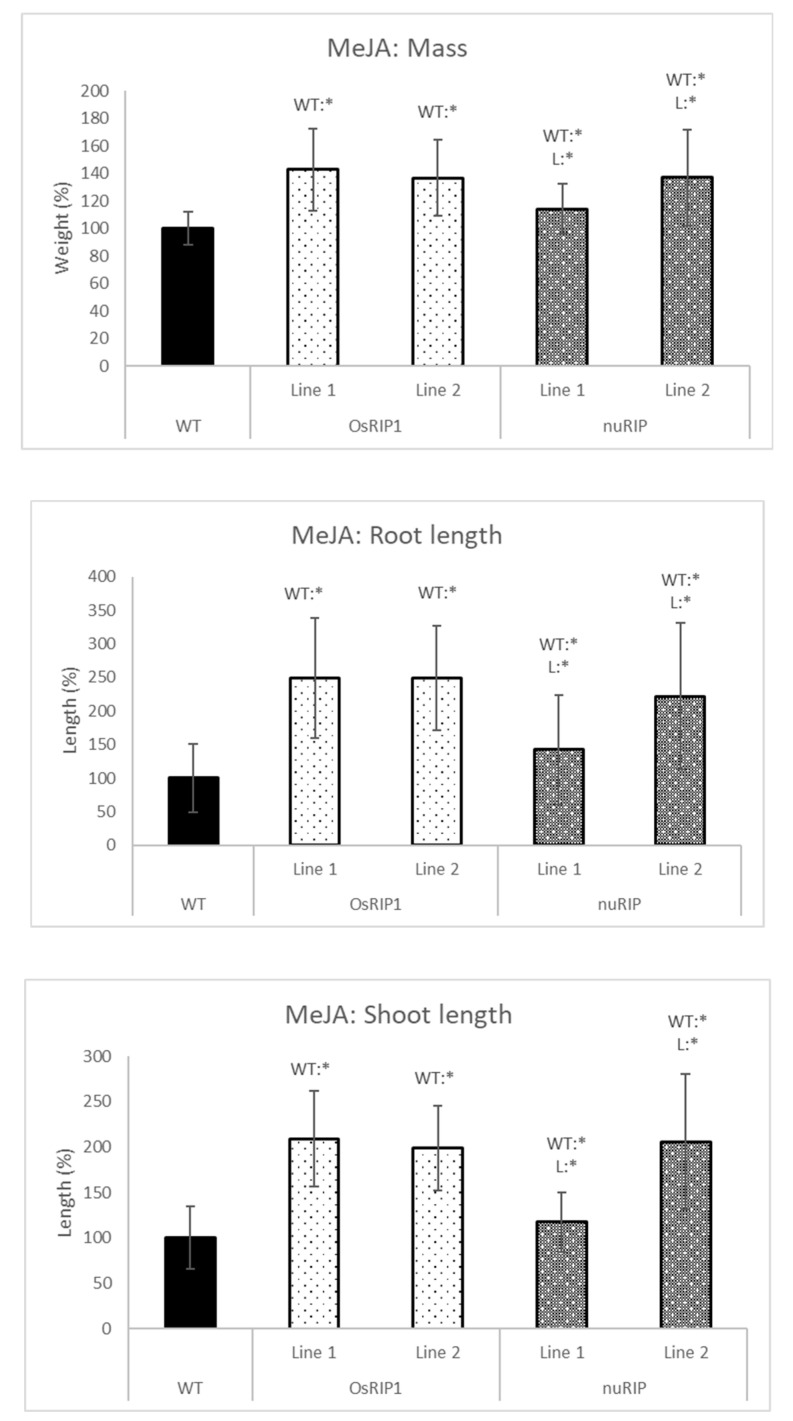
Total mass, shoot and root length of one-week-old plants from different transgenic lines subjected to 5 µM methyl jasmonate (MeJA) compared to those of wild-type. WT:* refers to a significant difference to wild-type plants. L:* indicates a significant difference to the other transgenic line of the same construct. The histogram is based on averages; error bars represent the standard deviation. Each data point is gathered from 50 plants per line.

The plant response to methyl jasmonate was measured by the addition of 5 µM MeJA to the growth solution (Figure 8). Transgenic seedlings overexpressing nuRIP or OsRIP1 all had a significantly higher mass, and they all had longer roots and shoots. For plants belonging to OsRIP1 line 1 and 2 and nuRIP line 2, the mass was around 1.4 times the mass of wild-type plants exposed to MeJA, while the shoots were twice as long as those from the wild-type plants and the roots were up to 2.5 times longer than those of the wild-type plants. Although the plants of nuRIP line 1 were significantly heavier and longer than wild-type plants, the differences with wild-type plants were smaller compared to the other three transgenic lines: 1.1 times the mass of wild-type plants, 1.2 times the shoot length and 1.4 times the root length.

**Figure 9:**
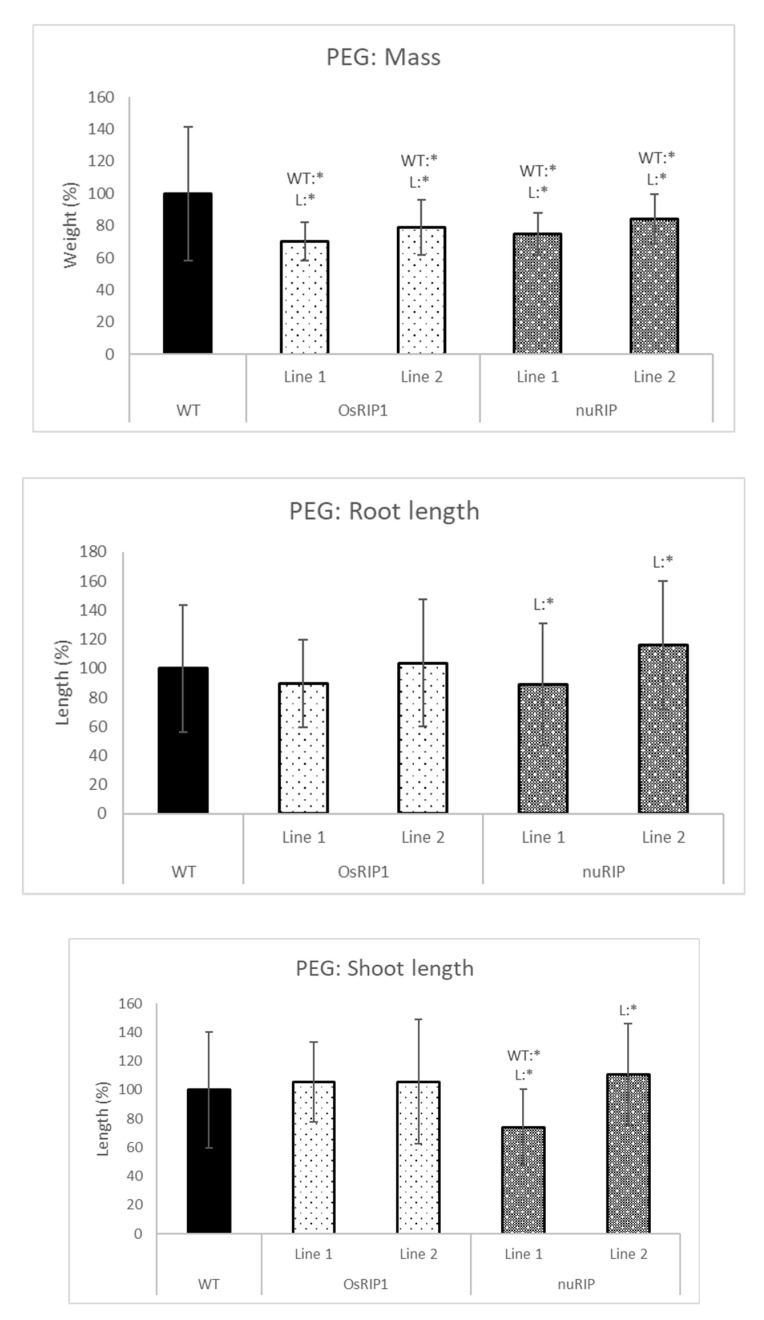
Total mass, shoot and root length of one-week-old plants from different transgenic lines subjected to 20% PEG-6000 compared to those of wild-type. WT:* refers to a significant difference to wild-type plants. L:* indicates a significant difference to the other transgenic line of the same construct. The histogram is based on averages; error bars represent the standard deviation. Each data point is gathered from 50 plants per line.

**Figure 10:**
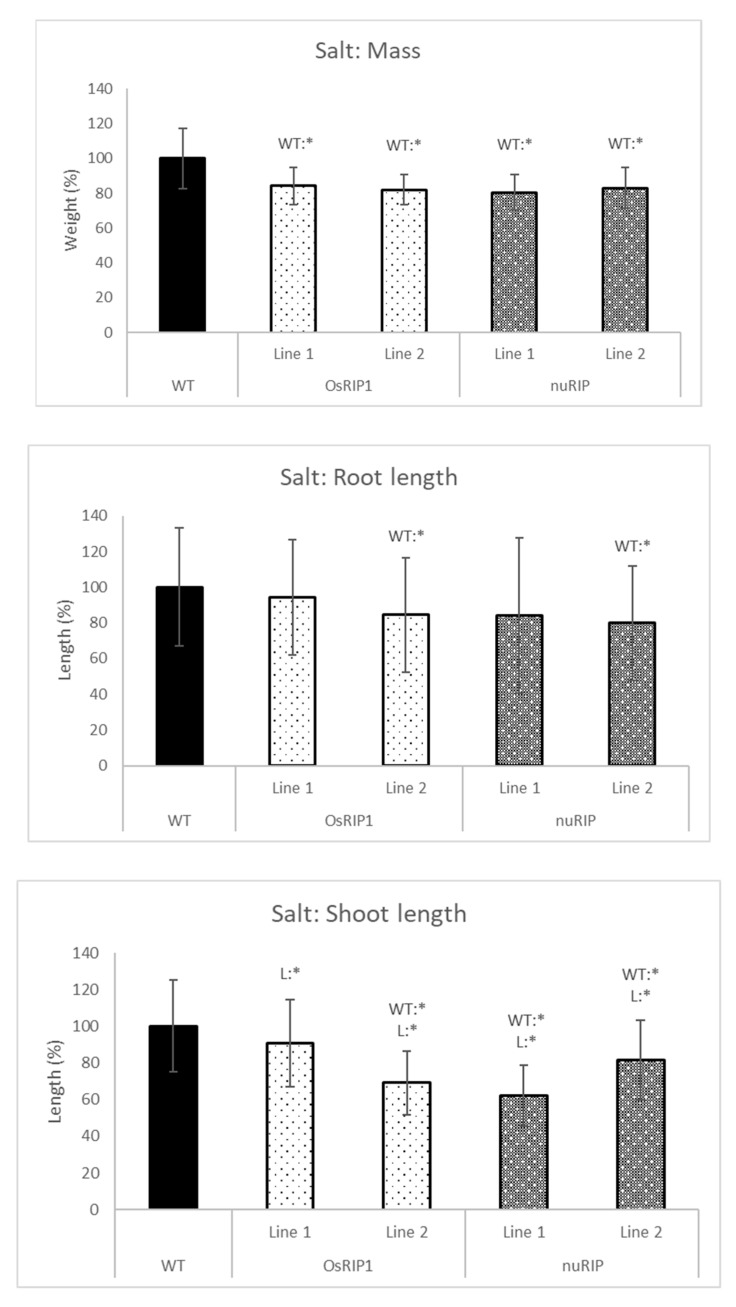
Total mass, shoot and root length of one-week-old plants from different transgenic lines subjected to 150 mM salt compared to those of wild-type. WT:* refers to a significant difference to wild-type plants. L:* indicates a significant difference to the other transgenic line of the same construct. The histogram is based on averages; error bars represent the standard deviation. Each data point is gathered from 50 plants per line.

The addition of 20% PEG to the hydroponic system mimicked drought stress (Figure 9). Overall, plants overexpressing OsRIP1 or nuRIP had a significantly lower mass than the wild-type plants. In addition, plants from nuRIP line 1 had a significantly shorter shoot than the wild-type plants. When salt stress was applied by the addition of 150 mM NaCl to the growth solution (Figure 10), rice plants overexpressing OsRIP1 and nuRIP revealed a lower mass than wild-type plants when subjected to salt stress. For plants from OsRIP1 line 2, the shoot and root length were lower than in the wild-type plants. Plants from both nuRIP lines had a significantly shorter shoot, whereas plants from line 2 also had a shorter root.

## 3. Discussion

In this study, two RIP sequences were selected from the extended family of rice RIPs. The OsRIP1 sequence has recently been reported as a type 1 RIP, and the recombinant protein made in *E. coli* was proven to be enzymatically active on ribosomes from rabbits and insects [10]. In contrast, nuRIP most probably encodes a type 3 RIP. At present, the enzymatic activity of nuRIP has not been proven. However, the presence of all active site residues at exactly the same position as in the RIP domain of the OsRIP1 sequence and previous three-dimensional modeling studies [6] all suggest that nuRIP is enzymatically active. Subcellular localization studies using eGFP fusion proteins for nuRIP have shown that this RIP locates to the cytoplasm as well as to the nucleus, similar to OsRIP1. This localization pattern is in agreement with the absence of a signal peptide, suggesting that the mRNA will be translated on free ribosomes in the cytoplasm. This is in contrast to most previously studied RIPs that classify as secretory proteins that end up in vacuoles or are secreted from cells [10]. The nuRIP sequence contained a nuclear localization sequence in its C-terminal sequence. To our knowledge, no other RIP sequences have been reported to contain a signal for transport to the nucleus.

While the OsRIP1 sequence encodes the typical polypeptide for a type 1 RIP, the nuRIP polypeptide is much larger and contains, in addition to the RIP domain, a significant stretch of amino acids at the C-terminus. The C-terminal domain of nuRIP shows no apparent homology to another protein domain. A high degree of sequence homology was detected between the nuRIP sequence and both domains in JIP60, a type 3 RIP composed of a RIP domain and a domain resembling the eukaryote translation initiation factor 4E. JIP60 is a unique RIP from barley, and its activity is based on the confirmation of the protein. The complete JIP60 will reversibly inactivate ribosomes by disassembling ribosomes into separate subunits. Subsequently, the JIP60 is processed into the two separate protein domains. The second domain can then recruit specific mRNA transcripts for translation [11]. We hypothesize that nuRIP from rice has similar activity to JIP60 from barley.

To unravel the physiological importance of OsRIP1 and nuRIP in rice, overexpression lines were created. Analysis of the shoot length at different time points during the development of the rice plant revealed that plants overexpressing nuRIP had a significantly reduced length, whereas the growth of rice lines overexpressing OsRIP1 was not significantly affected when grown in the absence of stress and in greenhouse conditions.

No significant differences between wild-type and transgenic plants were apparent at the final time point of analysis with regard to the total number of seeds, the 100-seed weight and the shoot weight. This could indicate that overexpression of the RIPs does not affect these parameters, but the high variability in the data could also be due to the promoter sequence used in these transgenic lines. In the pMBb7Fm21GW–UBIL vector, the expression of the transgene is driven by the UBIL-promoter. This promoter sequence is equivalent to the maize ubiquitin (Ubi1) promoter with the 5′UTR-intron still attached [12,13] and is frequently used in cereals, as it has been shown to be highly active in monocots. In maize protoplasts, the transient expression using this Ubi1-promoter showed a 10-fold higher expression than with the 35S promoter, and in rice protoplasts, a 13-fold higher expression was reported [14,15]. Although the promoter is active in many tissues, it is not a constitutive promoter. Promoter activity studies by Cornejo et al. using a GUS staining assay on transformed plants [16] showed that GUS staining was very intense in the young roots, but as these matured, the GUS expression decreased dramatically. Similarly, young leaves showed a very high GUS expression, but GUS expression decreased when plants aged. The somatic tissue of the anthers did not show any GUS staining, while pollen grains showed GUS staining in varying degrees. GUS expression was also found in the tissue culture phase. It is clear that the Ubi1-promoter is mostly active in dividing tissues and early in the development. Ubi1 promoter activity is greatly reduced when the tissue matures. During the late developmental stages of transgenic plants, it is possible that the expression of the transgene is very low. In wild-type rice, secreted RIPs are expressed mostly in the anthers [17]. It was shown that another anther-specific, secreted RIP in rice was expressed specifically in the tapetum [7,9]. However, the Ubi1 promoter is not active in the somatic cells in the anthers [16], meaning that there is probably no overexpression of the RIPs under study in the tapetum.

Seedlings of different transgenic lines were tested for their tolerance to drought and salt stress and their response to ABA and MeJA. These plant hormones show a complex interplay and play a role in the overall stress response and development. In general, two large groups of hormones can be distinguished: those hormones promoting growth (gibberellin, auxin), which are also susceptibility factors, and growth-repressing hormones (ABA, salicylic acid, jasmonates), which also function as resistance factors. RNA sequencing data revealed that the expression of different RIPs is affected when rice is grown in stress conditions. The expression of OsRIP1 changes when rice is subjected to osmotic stress, ABA, jasmonates, cadmium, cold, drought and flooding, while the expression of nuRIP changes only during cadmium stress, drought, flooding and osmotic stress [6].

Jasmonates are important for plant defense and key in the wounding response; they play a critical role in the response against herbivores and necrotrophic pathogens [18]. Jasmonate signaling is vital in plant defense against chewing insects but makes plants more susceptible to infestation by sucking insects, e.g., the brown planthopper *Nilaparvata lugens* [19]. Another role can be found in the development of plants, more specifically, senescence [20,21,22]. Other developmental roles are floral development and growth inhibition [22,23,24,25]. Jasmonates are implicated in many abiotic stresses as well, such as drought, osmotic stress, salt, heavy metals and cold/heat stress. Some authors hypothesize that the function of jasmonates in abiotic stress could be due to the jasmonate-mediated regulation of stomatal closure [26,27,28]. Other authors attribute the function of jasmonates in abiotic stress to a jasmonate-controlled regulation of the antioxidant defense system [29]. In Triticum estivum foliar application of jasmonate increased the activity and transcript levels of different antioxidant enzymes such as superoxide dismutase, ascorbate peroxidase and catalase [30].

When jasmonates are applied exogenously, seedling growth, in general, is inhibited, next to inhibition of root growth, leaf expansion and hypocotyl elongation [23,25,31,32]. These effects were also observed in our experiments. Wild-type plants showed a reduction in growth in the presence of MeJA. After treatment with MeJA, OsRIP1 and nuRIP overexpressing lines are significantly larger than the wild-type plants. The growth inhibition by MeJA has a clear role, which is making sure that plants use the available energy to survive (fighting off pathogens/surviving abiotic stress). OsRIP1 and nuRIP seem to hijack this response and make sure the plants keep growing. This response is advantageous when there is no danger, or the danger is very superfluous/insignificant.

Similar to MeJA, ABA is known to be involved in many plant physiological processes [33]. ABA is important in the synthesis of proteins for seed storage, seed desiccation tolerance, seed dormancy and inhibition of phase transitions (germination stage—vegetative stage—reproductive stage) [34,35]. Next to a role in development, the ABA concentration increased after abiotic stresses such as cold, drought and salt [36]. In response to drought stress, ABA levels can increase ten-fold or more within a few hours and decrease quickly upon rehydration [37]. The ABA response is very important for the plant despite the associated growth inhibition. ABA induces stomatal closure and reduces the canopy expansion [33]. Thanks to these effects of ABA, the water loss is reduced, and the plant can survive long periods of drought [33]. Furthermore, many genes for the biosynthesis of LEA-like proteins are upregulated by ABA. These osmolytes protect the plant against stress-induced damage [38,39]. ABA also plays a role in biotic stress. Stomatal closure due to ABA stops the entry of pathogens into the plant. In the later stages of infection, ABA mostly has a negative effect [40,41]. In this study, no clear benefits related to the overexpression of RIPs were detected during ABA treatment. The growth of both wild-type plants and transgenic plants was inhibited in the presence of ABA.

The research of Jiang et al. with overexpression lines of an anther-specific, secreted RIP (OsjRIP7.2) showed that overexpression of this RIP increased drought tolerance. The overexpression lines were treated with 30% PEG and, after two hours, showed no aberrant phenotype while wild-type plants were wilting. After 4 h of stress with 30%, PEG two week old seedlings were washed, and two weeks later, the transgenic lines showed a higher survival rate [8]. Jiang et al. [8] also reported better performance of transgenic RIP lines under salt stress. In contrast, our results revealed no beneficial effect of overexpressing RIPs. However, our experimental setup was slightly different. Jiang et al. used a higher salt concentration (200 mM) and assessed the effects after 8 h, whereas in this study, only 150 mM of salt was used, and the plant performance was evaluated after 3 days of stress. Another possibility is that different RIPs have evolved different roles and are involved in the plant response to variable stress responses.

For our experiments, we selected two transgenic lines with different overexpression levels of the RIPs under study. Using two lines with a very different overexpression level of OsRIP1 or nuRIP allowed us to study the effect(s) of overexpression and assess whether there is a dose-dependent effect of RIP overexpression. In this study, we observed that even though the overexpression levels for the RIPs are very different between lines, plants from both transgenic lines show a similar response.

Judging from our experiments, a clear effect of nuRIP and OsRIP1 overexpression was only observed after MeJA treatment. The reason for this specificity could be attributed to the ribosomes themself. There are different ways in which ribosomal proteins can affect the ribosomes [42]. The pool of ribosomes in a cell is highly heterogeneous. Ribosomes are made up of different ribosomal proteins, and for these proteins, multiple paralogs exist in a single organism [43,44,45]. These paralogs can be expressed at the same time or in response to certain environmental cues [45]. The genome of *Arabidopsis thaliana* has 429 genes encoding ribosomal proteins, and many of them show variation in the nucleotide sequence and have a different expression throughout development [46,47,48]. Similar to *Arabidopsis*, 123 genes encode for only 34 large ribosomal subunit proteins in rice [49,50]. Some of these genes were proven to be differentially regulated by abiotic stresses and hormones [51]. Another potential regulatory mechanism is the post-translational modification of ribosomal proteins. In yeast, ribosomal proteins were found to be methylated, acetylated and hydroxylated [52]. Similarly, in *A. thaliana*, many ribosomal protein families were proven to be covalently modified [53]. In Zea mays, UV-B exposure of leaves induced phosphorylation of a ribosomal protein [54]. The differences in the sequences of the paralogs or the modification of proteins could influence the interaction between RIPs and the ribosomes, and thus the RIP activity in the cell. Research in mammalian cell lines showed that ribosomes consisting of specific ribosomal protein paralogs have a preference for certain subsets of mRNAs [55]. The natural role of RIPs in the abiotic stress response of rice could be to attenuate the stress response. This means that after the stress has gone, RIPs orchestrate a very rapid change to normal growth by reprogramming the translation machinery through the inhibition of stress associated ribosomes and leaving only normal ribosomes intact with a preference for nonstress implicated mRNAs. Jasmonates are known to reprogram the translation machinery. It is possible that OsRIP1 and nuRIP specifically inhibit ribosomes associated with jasmonate associated changes and reprogram translation. It has been reported that JIP60 from barley can only act on ribosomes from leaves treated with MeJA or osmotically stressed/desiccated leaves [11,56]. NuRIP is a putative type 3 RIP, with high similarity to JIP60 [11,56], suggesting that nuRIP could have a similar activity in rice compared to JIP60 from barley. The RIP activity of OsRIP1 has experimentally verified on ribosomes from rabbit reticulocytes as well as ribosomes present in wheat germ extracts. It appears that although OsRIP1 inactivated wheat ribosomes, a tenfold increase in RIP concentration was necessary to obtain the same extent of inactivation as observed towards ribosomes from reticulocytes [10]. Although the activity of OsRIP1 towards ribosomes from rice has never been investigated, it is possible that OsRIP1 has evolved specificity to certain pools of ribosomes, such as ribosomes implicated in a particular stress response.

In this study, we have proven that RIPs can play a role in the plant response to abiotic stresses. Our experiments suggest that these non-secreted RIPs from the rice will most likely have an endogenous role in the cell. Bioengineers/biotechnologists/breeders could consider using RIPs to modulate the plant stress response (in the case of nuRIP and OsRIP1 overexpression, plants showed better growth despite the presence of MeJA). Studying the importance of genes by creating overexpression lines is only the first step in elucidating the physiological role of the proteins encoded by these genes. In the next step, knockout lines for the gene can be created. However, before we get there, we should also get some information about the expression of these genes in different tissues and how the expression changes in time and during the development of the plants. Only if we have all that information we can do targeted stress studies (check induction of expression by different stresses and hormones) at specific time points in the life cycle of the plant. Next, we should check the overexpression and knockout lines under these specific stress conditions. It should be mentioned that the construction of knockout lines (for nuRIP and OsRIP1) in rice may be challenging, taking into account that the rice genome harbors 38 closely related RIP genes [6]. This article represents the start of a journey to investigate the importance of OsRIP1 and nuRIP in the plant and how they can be used to advance agriculture all over the world. This article aims to spark interest and motivate researchers to study RIPs and their involvement in plant responses to abiotic stress.

## 4. Materials and Methods

### 4.1. Plant Material and Seed Sterilization

*Oryza sativa* ssp. Japonica cv. Nipponbare seeds were dehusked manually and incubated in 70% ethanol on a shaker (130 rpm) for 5 min, followed by 45 min in 5% NaOCl solution containing 0.01% Tween-20. Subsequently, the seeds were washed with autoclaved water at least eight times.

For seed multiplication, T1/T2/T3 seeds were sterilized as described above and incubated overnight in sterile water at 28 °C, 180 rpm. After blotting dry on sterile Whatman No 1 filter paper, seeds were incubated on seed germination medium (2.151 g/L MS basal salts, 0.56 mg/L Gamborg B5 vitamins, 30 g/L sucrose, 8 g/L agarose SPI, pH 5.8) containing 4 mg/L phosphinothricin (PPT) for 4 days under complete darkness, followed by 2 days under continuous light (200 µmol/m^2^/s) in a plant growth chamber at 30 °C with 85% relative humidity. Subsequently, plants were transferred into plastic pots (3 L) and grown in the greenhouse (26–28 °C) (UGent, Melle, Belgium).

### 4.2. Constructs

The open reading frame for OsRIP1 was amplified from the rice genomic DNA using the primers P2 and P3, while the nuRIP sequence was amplified from cDNA using the primers P4 and P5 (Supplementary Table S1). Polymerase chain reaction (PCR) was performed using Q5 high fidelity DNA polymerase according to the manufacturer’s instructions (NEB, Ipswich, MA, USA). While amplifying the OsRIP1, a high GC enhancer was added to the reaction. The amplified fragments were cloned into pJET1.2 vector using the CloneJet PCR Cloning kit (Thermo Fisher Scientific, Waltham, MA, USA). The plasmids were transferred into heat shock competent *Escherichia coli* TOP 10 cells, and the transformed bacterial colonies were selected on LB agar medium (Duchefa, Haarlem, the Netherlands) containing 0.2 mg/mL carbenicillin (Duchefa) and screened by colony PCR. Plasmid DNA was purified using the GeneJET plasmid miniprep kit (Thermo Fisher Scientific), and the sequence of OsRIP1 and nuRIP was verified through Sanger sequencing (LGC Genomics, Berlin, Germany).

The AttB sites required for Gateway^®^ Cloning (Life Technologies, Carlsbad, CA, USA) were added to the constructs in two consecutive PCR reactions. The first PCR used primer sequences complementary to the RIP sequence and contained part of the AttB sites (OsRIP1: P6–P7, nuRIP: P8–P9). In the second PCR reaction, the AttB sites were completed (P10–P11). PCR was performed with Q5 high fidelity DNA polymerase according to the manufacturer (NEB). For OsRIP1, the high GC enhancer was added to the PCR reactions. Subsequently, the PCR products were used as substrates in the BP recombination reaction with the pDONR221 vector (obtained from Plant Systems Biology, Vlaams Instituut voor Biotechnologie (VIB), Ghent, Belgium). Equimolar amounts of the PCR products with AttB sites and the donor vector were incubated overnight with the BP Clonase^®^ II enzyme mix (Thermo Fisher Scientific). Entry clones were transferred to *E. coli* TOP 10 cells. Transformants were grown on an LB agar medium containing 50 μg/mL kanamycin (Duchefa) and screened by colony PCR. The entry clones were purified using the GeneJET plasmid miniprep kit (Thermo Fisher Scientific) and sequenced by LGC Genomics with the DONR-F sequencing primer.

The pMBb7Fm21GW-UBIL vector (Plant Systems Biology (VIB)) was used to make the overexpression constructs. The T-DNA insertion region of this vector contained the bar gene under the control of the CaMV 35S promoter and nos terminator. The bar gene acted as a plant selection marker and conferred resistance to PPT. It also contained the maize ubiquitin promoter (UBIL promoter) to overexpress the gene of interest in rice.

The entry clones for the rice transformation were combined with the destination vector (pMBb7Fm21GW–UBIL vector) and incubated overnight with LR Clonase^®^ II enzyme mix (Thermo Fisher Scientific). The resulting expression clones were transferred into *Agrobacterium tumefaciens* strain EHA105 by electroporation, and the transformed bacterial colonies were selected on YEB agar medium (5 g/L beef extract (Lab M Ltd., Lancashire, UK), 5 g/L peptones (MP Biomedicals, Santa Ana, CA, USA), 1 g/L yeast extract (Duchefa), 5 g/L sucrose (Duchefa) with 10 mg/L rifampicin and 100 mg/L spectinomycin) (Sigma-Aldrich, Diegem, Belgium). The transformation was confirmed through colony PCR. Glycerol stocks were prepared and stored at −80 °C until further use.

### 4.3. Rice Transformation and Analysis of Transformed Plants

*Agrobacterium*-mediated genetic transformation of rice was performed as described by Lambin et al. [57]. Briefly, rice calli obtained from mature rice seeds were incubated for 10 min in *A. tumefaciens* strain EHA 105 suspension containing the pMBb7Fm21GW–UBIL vector and the construct. The infected calli were co-cultivated for 4 days on R2-COMAS medium and then incubated for 4 weeks on a selection medium containing 500 mg/L timentin and 50 mg/L PPT (Duchefa). The surviving calli were separated and incubated for 4 weeks on a pre-regeneration medium containing 300 mg/L Timentin and 30 mg/L PPT. Actively proliferating calli were collected from the pre-regeneration medium and then incubated for 6 weeks on the regeneration medium. The putatively transformed shoots were excised individually and rooted in the rooting medium. Well-rooted plantlets were transplanted into the plastic pots (3 L) containing soil and acclimatized, and hardened in the plant growth room and subsequently moved to the greenhouse. The plants were allowed to undergo self-pollination, seeds were harvested and designated as T1 seeds. A PCR reaction was performed on extracted genomic DNA for each transformant. The reaction was performed with Taq polymerase (VWR, Oud-Heverlee, Belgium), a forward primer binding on the UBIL promoter (Supplementary Table S1, primer: P1) and a reverse primer complementary to the C-terminal sequence of the open reading frame of each RIP sequence (Supplementary Table S1, OsRIP1: P3, nuRIP: P5). For the amplification of the OsRIP1 sequence, 6% DMSO and 1 M betaine were added to the reaction mixture. The amplified PCR products were separated by electrophoresis on a 1.5% agarose gel (Invitrogen, Carlsbad, CA, USA) in TAE buffer (20 mM Tris (MP Biomedicals), 0.5 mM EDTA (MP Biomedicals) and 5.71% glacial acetic acid (VWR)) and visualized with ethidium bromide (0.5 mg/mL). MassRuler DNA-loading dye (Thermo Scientific) and MassRuler DNA ladder mix were used (Thermo Scientific). Only those T0 plants which yielded a successful PCR reaction (and thus contained the transgene) were selected and propagated.

For each RIP gene, approximately 10 independent transgenic lines were generated. All these transgenic lines were screened for the expression of the respective RIPs and the presence of the resistance gene (to the herbicide phosphinothricin). Subsequently, all plants were transplanted to soil and grown in the greenhouse. After multiple generations, a selection was made for those lines that generated enough seeds. From the 3–4 lines that remained, two lines with very different overexpression levels were selected to assess whether there is a dose-dependent effect of the overexpression for each RIP under study. The plants used in the experiments are a mix of heterozygous and homozygous plants (second/third generation seeds) (Supplementary Table S2).

### 4.4. DNA Extraction

Plant material was frozen at −80 °C. After crushing in liquid nitrogen 1 mL CTAB buffer (2% CTAB, 0.1 M Tris/HCl pH 7.5; 1.4 M NaCl; 2 mM EDTA) was added per 100 mg plant material (from a single plant), followed by a chloroform:isoamyl alcohol (24:1) extraction. DNA was precipitated with 100% isopropanol and washed with 76% EtOH/0.2 M NaOCl and 76% EtOH/10 mM NH4OAc.

### 4.5. RNA Extraction and q-RT–PCR

Total RNA was isolated from 0.1 g leaf material (pooled from 10 plants) harvested from 6-week old T2 and T3 generation rice plants (grown hydroponically) using the spectrum plant total RNA kit (Sigma-Aldrich). After RNA extraction, a DNAse treatment was performed with DNAse I (Thermo Fisher Scientific). The quality of RNA was assessed with the NanoDrop 2000 spectrophotometer (Thermo Fisher Scientific). cDNA synthesis was performed using MMLV reverse transcriptase (Invitrogen, Carlsbad, CA, USA). The cDNA was diluted 2.5 times and used for PCR analyses.

Q-RT–PCR was performed with the 96-well CFX Connect™ real-time PCR detection system (Bio-Rad, Hercules, CA, USA) with iQ™ SYBR^®^ Green Supermix (Bio-Rad). A mix was generated with 1 µL of each primer (10 µM), 10 µL iQ™ SYBR^®^ Green Supermix, 2 µL cDNA and 6 µl of water. The Q-RT–PCR program was 10 min −95 °C, 41 cycles of 15 sec −95 °C, 25 sec −60 °C, 20 sec −72 °C. A melting curve was generated after every Q-RT–PCR run. Two biological and two technical replicates were used for each analysis. Three reference genes (EIF5C, EXP and EXPNar) were used for the normalization of the data (Supplementary Table S1, P16–P21) [58,59,60]. Melting curve analysis was performed using Bio-Rad CFX Manager 3.1 software. Reference gene stability and quality control of the samples were assessed using the qbase+ software from Biogazelle (geNorm M value <1, Coefficient of variation <0.5) [61]. Q-RT–PCR primers were made using the Primer3 software (http://biotools.umassmed.edu/bioapps/primer3_www.cgi). The specificity (BLAST search) and presence of secondary structures were analyzed in silico [62]. The specificity of primers was also verified by cloning and sequencing (OsRIP1: P12–P13, nuRIP: P14–P15 (Supplementary Table S1)). The analysis was done using the REST-384 software using the pairwise fixed reallocation randomization test (with 2000 randomizations) [63].

### 4.6. Hydroponic System for Growing Rice Seedlings

Sterilized seeds were incubated on a seed germination medium containing 4 mg/L PPT as indicated above. After incubation in complete darkness for four days followed by two days under continuous light (200 µmol/m^2^/s) in a plant growth chamber at 28 °C, plants were transferred to ½ Hoagland solution (2.5 mM KNO3, 0.5 mM KH2PO4, 2.5 mM Ca(NO3)2.4H2O, 1 mM MgSO4.7H2O, 14 µM H3BO3, 4 µM MnSO4.4H2O, 0.15 µM ZnSO4.7H2O, 0.015 µM (NH4)6Mo7O24.4H2O, 0.16 µM CuSO4.5H2O, 25 µM FeSO4.7H2O, 25 µM EDTA disodium salt.2H2O adjusted to a pH of 5.8). The ½ Hoagland solution was refreshed daily. Rice seedlings were grown in the hydroponic system for 10 days.

### 4.7. Phenotypic Analysis of Rice Plants

For phenotypic analysis of transgenic plants, two transgenic lines per RIP construct were selected. For each line, 15 plants were analyzed. T2/T3 seeds were sterilized as described above and germinated on seed germination medium containing 4 mg/L PPT. The seeds were incubated for four days in complete darkness, followed by two days under continuous light (200 µmol/m2/s) in a plant growth chamber at 30 °C with 85% relative humidity. Afterward, the plants were grown in the greenhouse (UGent, Melle, Belgium) at randomized positions (frequently changed) in standard potting soil. Pots (3 L) were irrigated with water every two hours (between 7.00 and 22.00). The young seedlings were irrigated twice with 200 mL of 6.5 mM FeSO4 and 6.8 mM (NH4)2SO4 in the first two weeks after transplanting to the soil. Temperatures in the greenhouse were generally between 26 and 28 °C during the day. Grow lights were switched on 12 h before sunset. If the temperature exceeded 32 °C, the greenhouse was ventilated (with outside air). A shading screen was deployed in the winter and autumn after sunset and opened at sunrise. In spring and summer, the screen opens at 7.00 and closes at 19.00.

The shoot length of the plants was measured at multiple time points (18, 30, 50, 78 and 119 days after germination). The shoot length was defined as the length from the bottom of the shoot until the end of the longest leaf. At 112 days after germination, the watering of the plants was stopped. After reproduction and ripening (119 days after germination), shoot mass and shoot length were measured, and seeds were harvested. The total amount of seeds and the 100-seed weight (without the pericarp) were calculated after the seeds were dried at 30 °C for four days. The flowering (heading date) was assessed at 78, 92, 112 and 119 days after germination. The day at which plants showed the presence of panicles was defined as the heading date. Statistics were done with Python 3.7 and the scipy.stats module (Anaconda Distribution 2019.1, https://www.anaconda.com/distribution/). Normality was assessed with the Shapiro–Wilk test. To test homogeneity of variances, the Bartlett (less sensitive to deviations from normality) and Levene test were performed. Differences between groups were assessed with the nonparametric Kruskal–Wallis test and the Wilcoxon rank-sum test (due to the non-normality of the data). In post hoc tests, multiple testing correction was performed according to the Bonferroni correction (*p*-value < 0.05). For consistency, the wild-type was divided into two groups and designated as line 1 and line 2 for the complete duration of the experiment and data analysis. Overall, it was observed that the two wild-type “lines” are practically identical, and for comparative analyses with overexpression lines were merged together.

### 4.8. Stress Assays

Stress assays were performed with rice seedlings grown on MS plates (2.3 g/L MS medium including vitamins (Duchefa), 15 g/L sucrose, 8 g/L agarose SPI, pH 5.8) containing 4 mg/L PPT (for transgenic lines). Four days after germination, seedlings were transferred to sterile filter paper and liquid MS medium (2.3 g/L MS medium including vitamins, 15 g/L sucrose, pH 5.8). Stress assays were performed using 5 µM MeJA (Sigma-Aldrich), 2 µM ABA (Sigma-Aldrich), 150 mM salt (Carl Roth) or 20% PEG-6000 (VWR). The concentrations of the stressors were based on literature and optimization [64,65,66,67,68,69,70]. Each treatment consisted of 50 germinated 4-day old seedlings. Each stress treatment lasted for 72 h. Afterward, plants were harvested. Total plant mass was assessed by weighing, and pictures were taken from the plants. The length of roots and shoots was measured using Fiji (ImageJ, https://fiji.sc/, [71]). Statistics were done with Python 3.7 and the scipy.stats module (Anaconda Distribution 2019.1, https://www.anaconda.com/distribution/). Due to the high number of plants per data point, the central limit theorem allows the use of parametric statistics (this was confirmed with a permutation test). Differences between groups were assessed with a t-test.

Seeds from T2 and T3 generation were used for all stress experiments (Supplementary Table S2). Fifty seedlings were used per line. Supplementary Table S2 shows the rice lines used in the different experiments.

### 4.9. Tobacco Infiltration and Microscopy

The coding sequence for the RIP was cloned after the 35S promoter in the expression vectors pK7WGF2 and pK7WFG2 to create the N- and C-terminal EGFP fusion constructs, respectively. The cloning started by adding attB sites to the coding sequence by PCR (to create the C-terminal fusion with eGFP, the stop codon was removed) followed by BP reaction into pDONR221 plasmid to end with the destination vector after LR reaction (as described above). All primers used for the construction of the vectors are described in Supplementary Table S1. A binary vector expressing free eGFP (pK7WG2 vector) was used as a control in the localization study. Transient transformation of tobacco and microscopic analyses were essentially performed as described by Lambin et al. [57]. Two days after injection of the epidermal cells, the infiltrated leaf area was analyzed under the Nikon A1R confocal laser scanning microscope (Nikon instruments). EGFP was excited with a 488 nm argon-ion laser, PI was excited with a 560 nm laser, and DAPI was excited with a 404 nm laser. Emission filters were 500–550 nm for EGFP, 570–620 nm for PI and 425–475 nm for DAPI. Image analysis was performed in ImageJ [71].

### 4.10. Sequence Analysis

The RIP sequences encoding OsRIP1, nuRIP and JIP60 were analyzed for the prediction of protein domains using NCBI Conserved Domains [72]. In addition, the sequences were investigated for the presence of a signal peptide (http://www.cbs.dtu.dk/services/SignalP/) or a nuclear localization signal [73]. Sequence alignments were performed with Clustal Omega [74].

## Data Availability

Data are available with authors.

## Supplementary Materials

Supplementary materials can be found at https://www.mdpi.com/1422-0067/22/3/1434/s1.

## Abbreviations

ABA	Abscisic acid
MeJA	Methyl jasmonate
PCR	Polymerase chain reaction
PPT	Phosphinothricin
qRT–PCR	Quantitative real-time polymerase chain reaction
RIP	Ribosome-inactivating protein
