# Supplementary material for: Effect of RIP Overexpression on Abiotic Stress Tolerance and Development of Rice
Source: Int J Mol Sci. 2021 Feb 1;22(3):1434. doi: 10.3390/ijms22031434 (PMC7867109; doi:10.3390/ijms22031434)
Supplement: Supplementary file 1 [file ijms-22-01434-s001.pdf]

Supplementary data

Supplementary Table 1: List of primers used

		Primer	*Description*
P1	UBIL-prom	TGGATGATGGCATATGCAGCAG	Forward primer UBIL promoter
P2	ORF OsRIP1	ATGGCGTTGAACCCGCT	Forward primer ORF OsRIP1
P3	ORF OsRIP1	TCAGTTCCCATGAAACAGCTGAAGC	Reverse primer ORF OsRIP1
P4	ORF nuRIP	ATGCATGGATCGAACCGAAGAAAATTGG	Forward primer nuRIP (ORF)
P5	ORF nuRIP	TCAAAAGAAGTCCGGCGACCAG	Reverse primer nuRIP (ORF)
P6	AttB-OsRIP1	AAAAAGCAGGCTTCACCATGGCGTTGAACCCGCT	Forward primer for AttB1-site OsRIP1
P7	AttB-OsRIP1	AGAAAGCTGGGTGTCAGTTCCCATGGTGGAACAGCTGG	Reverse primer for AttB2-site OsRIP1
P8	AttB- nuRIP	AAAAAGCAGGCTTCACCATGCATGGATCGAACCGAA	Forward primer for AttB1-site nuRIP
P9	AttB- nuRIP	AGAAAGCTGGGTGTCAAAAGAAGTCCGGCGACCAG	Reverse primer for AttB2-site nuRIP
P10	AttB1	GGGGACAAGTTTGTACAAAAAAGCAGGCT	Forward primer rest of AttB1-site
P11	Att2	GGGGACCACTTTGTACAAGAAAGCTGGGT	Reverse primer rest of AttB2-site
P12	OsRIP1	CTGCTGTTCGTCGAGAAGGT	Forward qPCR primer (OsRIP1)
P13	OsRIP1	TCTACCAGCAGCAGCAACTT	Reverse qPCR primer (OsRIP1)
P14	nuRIP	GCCGACGATTAGTGGAGTTG	Forward qPCR primer (nuRIP)
P15	nuRIP	GCGTTGAGAGTCGTGGATG	Reverse qPCR primer (nuRIP)
P16	EXP	TGTGAGCAGCTTCTCGTTTG	Forward qPCR primer to amplify a reference gene EXP: LOC_Os03g27010 (rice)
P17	EXP	TGTTGTTGCCTGTGAGATCG	Reverse qPCR primer to amplify a reference gene EXP: LOC_Os03g27010 (rice)
P18	EIF5C	CACGTTACGGTGACACCTTTT	Forward qPCR primer to amplify a reference gene EIF5C: LOC_Os11g21990.1 (rice)
P19	EIF5C	GACGCTCTCCTTCTTCCTCAG	Reverse qPCR primer to amplify a reference gene EIF5C: LOC_Os11g21990.1 (rice)
P20	EXPNarsai	AGGAACATGGAGAAGAACAAGG	Forward qPCR primer to amplify a reference gene EXPnarsai: LOC_Os07g02340.1 (rice)
P21	EXPNarsai	CAGAGGTGGTGCAGATGAAA	Reverse qPCR primer to amplify a reference gene EXPnarsai: LOC_Os07g02340.1 (rice)
P22	AttB-OsRIP1(C)	AGAAAGCTGGGTGTCAGTTCCCATGAAACAGCTGAAGC	Reverse primer for AttB2-site OsRIP1 without stop codon
P23	AttB-nuRIP(C)	AGAAAGCTGGGTGAAAGAAGTCCGGCGACCAG	Reverse primer for AttB2-site nuRIP without stop codon

Supplementary Table 2: Transgenic lines used in different stress experiments.

Treatment	*OsRIP1*	*nuRIP*
ABA	Line 1 (T3 generation)	Line 1 (T3 generation)
	Line 2 (T2 generation)	Line 2 (T3 generation)
Drought	Line 1 (T2 generation)	Line 1 (T3 generation)
	Line 2 (T2 generation)	Line 2 (T3 generation)
Salt	Line 1 (T2 generation)	Line 1 (T3 generation)
	Line 2 (T2 generation)	Line 2 (T3 generation)
MeJA	Line 1 (T3 generation)	Line 1 (T3 generation)
	Line 2 (T2 generation)	Line 2 (T3 generation)

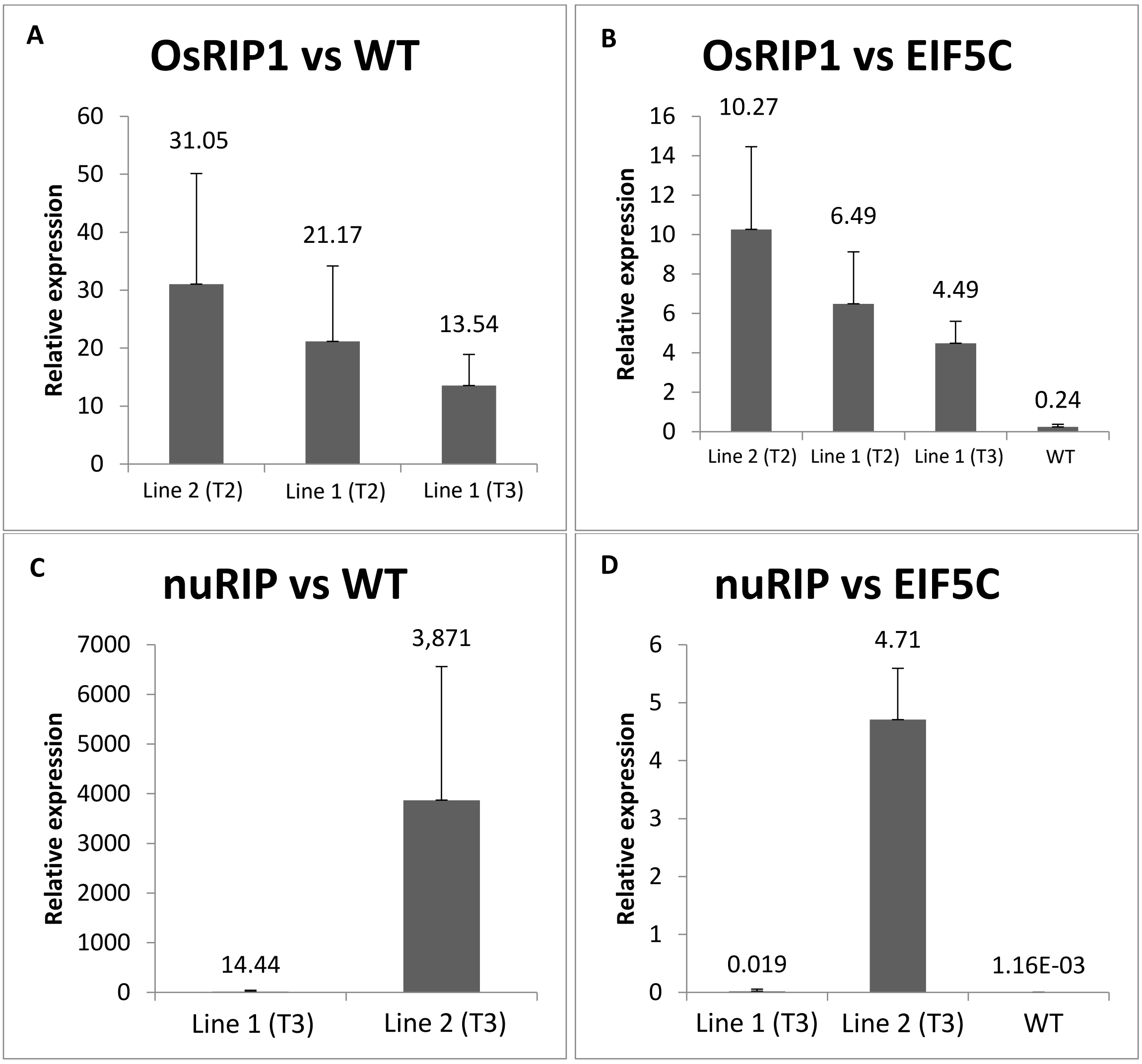

Supplementary Figure 1: Transcript levels for OsRIP1 and nuRIP in 10-day old seedlings determined by qRT-PCR. Panels A and C show the transcript levels for OsRIP1 and nuRIP in transgenic lines compared to the transcript levels in wild type plants. In panels B and D the transcript levels for OsRIP1 and nuRIP in transgenic lines and wild type plants are compared to the transcript levels for the housekeeping gene EIF5C.

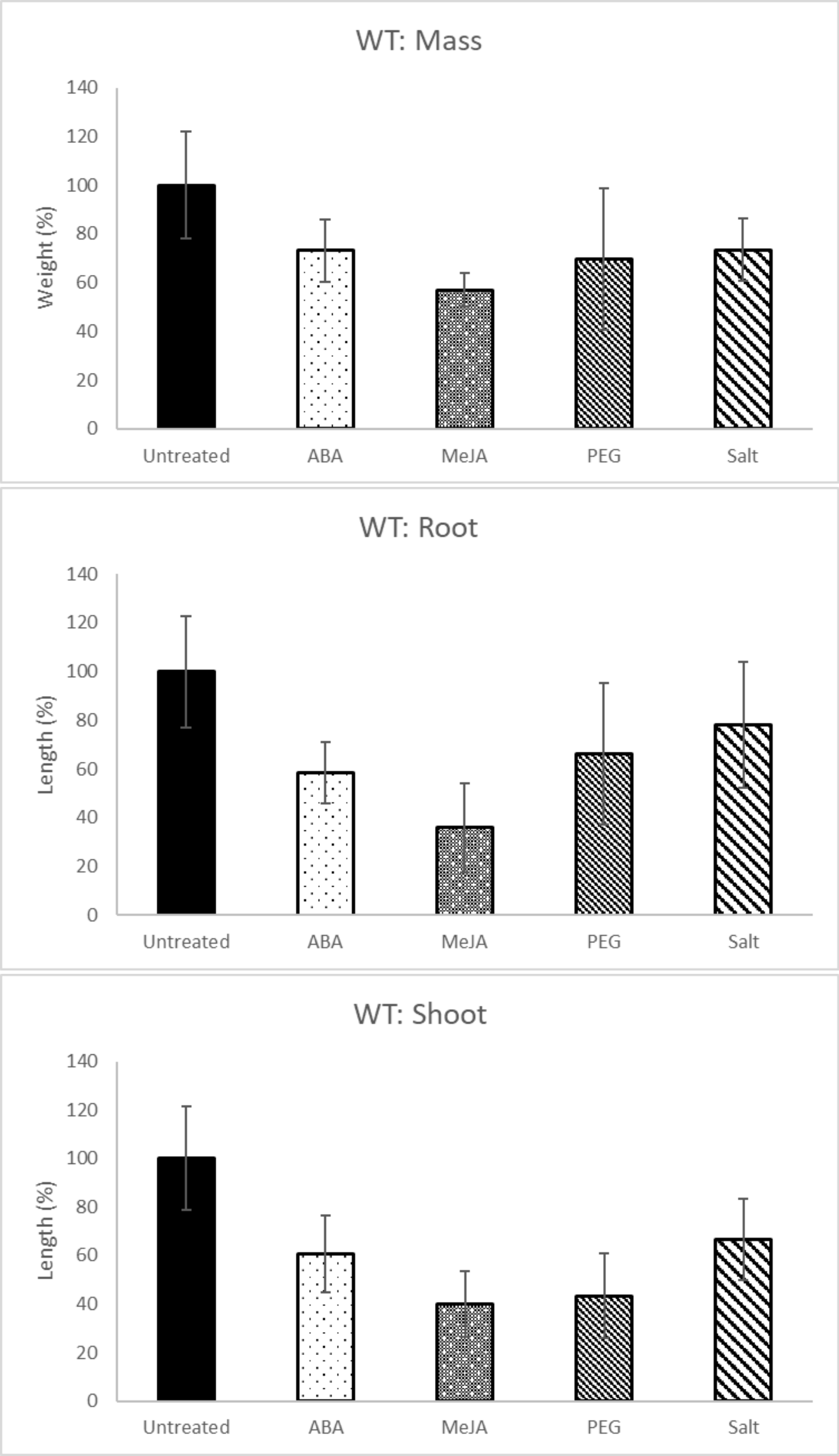

Supplementary Figure 2: Total mass, shoot and root length of one-week old wild type plants. The histogram is based on averages, error bars represent standard deviations. Each data point is gathered from 50 plants per line. No statistical differences were calculated because the untreated and treated plants were grown at different timepoints.
